# Full Thickness Prophylactic Scleral Windows Decrease the Rate of Choroidal Effusion and Drainage Surgery after Ahmed Glaucoma Valve Implantation

**DOI:** 10.18502/jovr.v19i1.15419

**Published:** 2024-03-14

**Authors:** Afsoon Baghbanmanesh, Masoumeh Sadat Masoumpour, Reza Razeghinejad

**Affiliations:** ^1^Poostchi Ophthalmology Research Center, Department of Ophthalmology, School of Medicine, Shiraz University of Medical Sciences, Shiraz, Iran; ^2^Wills Eye Hospital, Philadelphia, PA

**Keywords:** Glaucoma, Choroidal Effusion, Ahmed Glaucoma Valve

## Abstract

**Purpose:**

To evaluate the effect of creating a full-thickness prophylactic scleral window (PSW) during Ahmed glaucoma valve (AGV) surgery on the rate of postoperative choroidal effusion and choroidal drainage surgery.

**Methods:**

In this retrospective matched case-control study, after insertion of AGV tube a PSW was created in one group. The control-matched group had AGV without a PSW. Primary outcome measures were the rate of choroidal effusion formation and choroidal drainage surgery.

**Results:**

In total, 544 patients (604 eyes) had undergone AGV implantation from 2013 to 2017. The PSW group consisted of 111 eyes of 111 patients and the control group had 111 eyes of 98 matched patients. There were no differences for systemic diseases, number of anti-glaucoma drugs, aspirin use, smoking, laterality of the operated eye, axial length, and central corneal thickness between the groups. Out of 12 eyes with choroidal effusion, only one belonged to the PSW group (P=0.02). Six eyes in the control group needed choroidal drainage surgery, but none of the eyes in the PSW group required this procedure (P=0.02). No intra- and postoperative issues were observed at the site of the scleral window in the PSW group.

**Conclusion:**

PSW creation during AGV surgery is a safe method to decrease the rate of choroidal effusion and choroidal drainage surgery.

##  INTRODUCTION

Glaucoma is one of the leading causes of irreversible visual impairment. A variety of surgical techniques including Ahmed glaucoma valve (AGV) implantation are employed to lower Intraocular pressure (IOP) and stop or delay progressive visual loss.^[1]^ Choroidal effusions is one of the intra- or postoperative complications of filtration surgeries.^[2]^ The reported incidence of choroidal effusion by clinical examination following glaucoma drainage devices is 11.7 to 15%.^[3,4,5]^ However, the incidence has been as high as 35.1% detected in wide-field fundus photography.^[6]^ The majority of focal and limited effusions resolve with observation and medical therapy. However, a large or long-standing effusion is associated with visual loss due to the changes in lens or intraocular lens position and chorioretinal tissues.^[7,8,9]^ Indications for surgical management of the choroidal effusion include the presence of a grade 3 shallow anterior chamber (lens-cornea touch), and kissing, or persistent choroidal effusion.^[10,11]^ Instilling viscoelastic agents in the anterior chamber and tube ligature have been recommended to decrease the rate of shallow anterior chamber and choroidal effusion.^[12]^ 13Previous studies showed that prophylactic scleral window (PSW) in trabeculectomy was an effective method to prevent choroidal effusion or hemorrhage.^[14,15,16]^ To the best of our knowledge, there is no prior report on the effect of the PSW on the rate of choroidal effusion and choroidal drainage surgery after AGV implantation. In this study, we evaluated the effect of PSW on the rate of choroidal effusions and choroidal drainage surgery with AGV implantation.

##  METHODS

This retrospective, matched case-control study was conducted in a tertiary care eye hospital. We reviewed the chart of the patients who had AGV surgery between March 2013 and February 2017. The study protocol was reviewed and approved by the local ethical committee at Shiraz University of Medical Sciences, Shiraz, Iran; and followed all relevant tenets of the Declaration of Helsinki. The approval number is IR.SUMS.MED.REC.1398.419.

**Figure 1:**
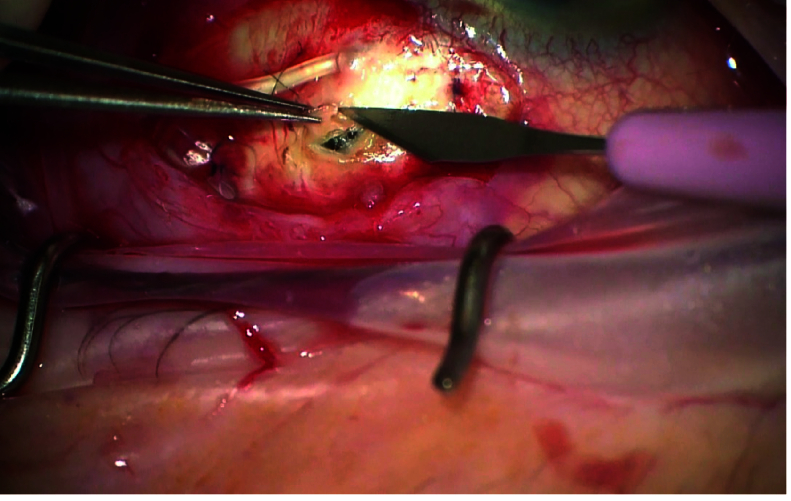
Creation of prophylactic scleral window during Ahmed glaucoma valve implantation.

All patients who had AGV with PSW were included. The control group was selected among those who had AGV without any PSW and matched with the PSW group for age, axial length, and type of glaucoma, systemic diseases, and standalone AGV or combined with phacoemulsification. All surgeries were performed by two glaucoma specialists using the same technique. After applying a corneal traction suture, a fornix-based conjunctival peritomy was performed. The primed AGV (model FP7, New World Medical, Rancho Cucamonga, LA, USA) was sutured to sclera 8-12 mm posterior to the limbus. After filling the anterior chamber with a dispersive viscoelastic agent, the trimmed tube was inserted into the anterior chamber through a tunnel created by a 23-gauge needle. After fixating the tube to the sclera with a 10.0 nylon suture, it was covered with a scleral patch. The borders of the scleral patch were loosely sutured. No tube ligation was done. Then the conjunctiva was closed with 8.0 vicryl sutures. In the PSW group a full thickness triangular shaped scleral window with the dimensions of 1 mm was created 4-5 mm posterior to the limbus next to the tube portion of the AGV and then the triangular flap was excised to leave the window open before closing the conjunctiva. An ophthalmic viscosurgical device (OVD ) was at the end of surgery in both groups (Figure 1 and Video 1).

Demographic data, medical and past ocular history, smoking status, preoperative visual acuity, IOP, number of glaucoma medications, axial length, central corneal thickness, the first month postoperative complications including choroidal effusion, suprachoroidal hemorrhage, tube cornea touch, diplopia, aqueous misdirection, hyphema, endophthalmitis and the surgical interventions for management of complications were collected. Statistical analysis was performed using SPSS version 22. Chi-square test and Fisher's exact test were used to compare categorical variables and T-test and Mann Whitney u test for numerical values. A P value less than 0.05 was considered significant.

**Table 1:** Demographic and preoperative characteristics of prophylactic scleral window and control groups.

	**Prophylactic scleral window group**	**Control group**	***P*** **-value**
Sex (male/female)	69/42	62/49	0.339 ${\;}^{a}$
Age (mean ± SD)	45.3 ± 26.8	51 ± 22.3	0.22 ${\;}^{d}$
Laterality (right/left)	53/58	65/46	0.107 ${\;}^{a}$
Diabetes mellitus	16	24	0.162 ${\;}^{a}$
Systemic hypertension	14	14	1 ${\;}^{a}$
Asthma	0	3	0.247 ${\;}^{b}$
Cerebrovascular accident	0	2	0.498 ${\;}^{b}$
Myocardial infarction	3	2	1 ${\;}^{b}$
Aspirin	9	9	1 ${\;}^{a}$
Smoking	3	4	1 ${\;}^{b}$
Preoperative vision LogMAR (mean ± SD)	3.2 ± 2.9	3.4 ± 2.6	0.62 ${\;}^{d}$
IOP (mean ± SD) mmHg	28.4 ± 9.1	30.4 ± 9.4	0.116 ${\;}^{c}$
Cup disc ratio (mean ± SD)	92.5 ± 19.7	90.6 ± 23.5	0.732 ${\;}^{d}$
Number of glaucoma medications (mean ± SD)	3.7 ± 1.08	3.9 ± 0.95	0.225 ${\;}^{d}$
Axial length (mean ± SD) mm	23.19 ± 1.6	23.18 ± 1.5	0.976 ${\;}^{c}$
Central corneal thickness (mean ± SD) µ	542.6 ± 63.1	536.1 ± 44.7	0.638 ${\;}^{c}$
	
	
SD, standard deviation ${\;}^{a}$ Chi square test; ${\;}^{b}$ Fisher's exact test; ${\;}^{c}$ T-test; ${\;}^{d}$ Mann–Whitney U-test			

**Table 2:** Types of glaucoma in the prophylactic scleral window and control groups.


**Types of glaucoma**	**Prophylactic scleral window group**	**Control group**
Neovascular glaucoma	19	21
Open-angle glaucoma with failed prior trabeculectomy	23	12
Primary angle closure glaucoma	12	18
Glaucoma following penetrating keratoplasty and pars plana vitrectomy	12	17
Congenital glaucoma	13	7
Aphakic glaucoma	9	8
Pseudoexfoliative glaucoma	7	7
Traumatic glaucoma	6	7
Uveitic glaucoma	2	4
Iridocorneal endothelial syndrome	1	1
Anterior segment dysgenesis	1	0
	
	
*P*-value of Fisher's exact test: 0.57		

**Table 3:** Early postoperative complications after Ahmed glaucoma valve surgery in the prophylactic scleral window and control groups.


	**Prophylactic scleral window group**	**Control group**	***P*** **-value ${\;}^{a}$ **
Tube corneal touch	0	1	1
Diplopia	0	1	1
Suprachoroidal hemorrhage	0	1	1
Aqueous misdirection	3	2	0.64
Choroidal effusion	1	11	0.02
	
	
${\;}^{a}$ Fisher's exact test			

##  RESULTS

Charts of 544 patients (604 eyes) were reviewed. One hundred eleven eyes of 111 patients had received AGV and PSW. Among 433 patients (493 eyes) without PSW, 98 patients (111 eyes) who matched the PSW group were included as the control group.

There were no differences between the groups for the sex, age, systemic diseases, aspirin use, number of glaucoma medications, smoking, type of glaucoma, laterality of the operated eyes, preoperative IOP and visual acuity (VA), axial length, and central corneal thickness. [Table 1 and Table 2].

Eighteen eyes in the PSW group and 19 eyes in the control group underwent combined phaco-AGV implantation. Eight eyes received intravitreal bevacizumab; four of them belonged to the PSW group ( P = 0.961)

Early postoperative complications are presented in Table-3. There was no statistically significant difference between both groups in terms of complications except for choroidal effusion. One eye in the PSW group and 11 eyes in the control group developed choroidal effusion ( P = 0.02).

None of the eyes in the PSW group needed choroidal drainage surgery, but 6 eyes of the control group underwent choroidal drainage surgery ( P = 0.02). Two eyes in the PSW and 3 eyes in the control group required second shunt implantation ( P = 0.62).

##  DISCUSSION

Choroidal effusion is one of the bothersome complications of AGV surgery for both patients and physicians.^[17]^ In the current study, the incidence of choroidal effusion was 9.91% and 0.9% in the control and PSW groups, respectively. PSW significantly decreased the rate of choroidal effusion formation and choroidal drainage surgery. None of the eyes of the PSW group needed choroidal drainage surgery.

The reported incidence of choroidal effusion following AGV implantation is between 11.7 and 35.1%.3-6 The incidence of 35.1% was reported in a study using wide-field fundus photography. By drawing a virtual circle presenting 45-degree fundus photography, the incidence decreased to 16.9%.^[6]^ Shin et al assessed the risk factors of choroidal effusion following AGV implantation in a retrospective case-control study. They showed that age, central corneal thickness, axial length, type of glaucoma, history of combined cataract with glaucoma surgery, systemic hypertension, diabetes mellitus, and severity of the visual field defect were different between choroidal effusion and non-choroidal effusion groups. In a multivariate analysis, age, pseudoexfoliative glaucoma, pseudophakia, and systemic hypertension were risk factors of choroidal effusion.^[6]^

Some factors have been recommended to be taken into account to decrease the chance of effusion. Lowering the IOP before surgery, having well-controlled blood pressure, avoiding sudden IOP drop, and instilling viscoelastic agents in the anterior chamber are among the recommended factors. If lowering the IOP with medications in the preoperative period is not feasible, reduction of IOP by creating a paracentesis at the beginning of surgery is one of the recommended methods to avoid sudden IOP drop. Instilling viscoelastic agents in the anterior chamber and stopping aqueous suppressant medications few days before surgery may reduce the risk of early postoperative hypotony and choroidal effusion.^[18]^ The patients may be advised to avoid straining and Valsalva-type maneuvers in the early postoperative period. However, we as physicians may not be able to stop the aqueous suppressants before surgery or the patients may not follow the postoperative instructions. Creating the PSW can decrease the chance of choroidal effusion and re-operation. The prophylactic scleral window will not decrease the chance of choroidal effusion formation, but it drains any formed effusion and decreases the risk of kissing choroidal effusion and flat anterior chamber. A retrospective case series on prophylactic sclerotomy during the standard trabeculectomy on 33 eyes of 28 juvenile open-angle glaucoma and 15 eyes of 12 primary congenital glaucoma patients resulted in a complete success rate of 75.8%, and the qualified success rate of 90.0% at 3 years. One eye had intraoperative expulsive hemorrhage and two had delayed expulsive hemorrhage, none of them needed any further surgery and their vision was not affected.^[14]^

Babushkin et al reported a 2.3 times reduction (28.1 to 12.1%) in the incidence of choroidal effusion following trabeculectomy in those who received prophylactic scleral window.^[15]^ They also reported that superior double cross-like sclerotomy during trabeculectomy is an effective method for the prevention of choroidal effusion. The incidence of effusion was 8 times lower (26.7%versus 3.3%) in the sclerostomy group.^[16]^ To the best of our knowledge, there is no prior report evaluating the role of PSW in AGV implantation.

In a retrospective study, the AGV tube ligation was compared with no-ligation. The success rate at 12 months was similar between both groups (P *= *0.84). The rate of complications related to low postoperative IOP (i.e., ocular hypotony, shallow anterior chamber, and choroidal effusion) was lower in tube ligature group. The incidence of mentioned complications was as low as 1%, 0% and 4 % in the ligated AGV group as compared with 11%, 8%, and 6% in the non- ligated AGV group.^[13]^ Tube ligature however, may be associated with transient IOP spike which may not be safe in patients with advanced glaucoma.

Choroidal effusion associated with grades 1 and 2 shallow anterior chamber may be treated with topical/oral steroids and cycloplegics. Drainage of choroidal effusion is indicated in cases with kissing choroidal detachment, grade 3 shallow anterior chamber and protracted effusion with grade 1 and 2 shallow anterior chamber.^[10]^ None of the patients in the PSW group of our study needed drainage surgery while 6 in the control group.

The limitations of our study include that of being a retrospective study and lack of any imaging for documenting the choroidal effusion. However, inclusion of more than one hundred cases and diverse types of glaucoma in each group are the strengths of the current study.

In summary, PSW is a safe method to decrease the rate of choroidal effusion following AGV implantation where the formed effusions did not need choroidal drainage and were managed medically.

##  Financial Support and Sponsorship

None.

##  Conflicts of Interest

None.
